# Effectiveness of a long-lasting insecticide treatment kit (ICON® Maxx) for polyester nets over three years of household use: a WHO phase III trial in Tanzania

**DOI:** 10.1186/s12936-021-03871-3

**Published:** 2021-08-19

**Authors:** Patrick K. Tungu, Wema Sudi, William Kisinza, Mark Rowland

**Affiliations:** 1 Amani Medical Research Centre, National Institute for Medical Research, Muheza, Tanzania; 2 Pan-African Malaria Vector Research Consortium (PAMVERC), PO Box 81, Muheza, Tanga, Tanzania; 3 London School of Hygiene and Tropical Medicine, London, UK

**Keywords:** Long-lasting insecticide treatment kits, LLIN, Lambda-cyhalothrin, *Anopheles gambiae*

## Abstract

**Background:**

ICON® Maxx (Syngenta) is an insecticide treatment kit of pyrethroid and binding agent for long-lasting treatment of mosquito nets. Interim recommendation for use on nets was granted by the World Health Organization (WHO) after successful evaluation in experimental huts following multiple washes. A full WHO recommendation is contingent upon demonstration of continued bio-efficacy after 3 years of use.

**Methods:**

A household-randomized prospective study design was used to assess ICON Maxx-treated nets over 3 years in north-eastern Tanzania. Conventional treated nets (with lambda-cyhalothrin, but without binder) served as a positive control. At 6-monthly intervals, cross-sectional household surveys monitored net use and physical integrity, while cone and tunnel tests assessed insecticidal efficacy. Pyrethroid content was determined after 12 and 36 months. A parallel cohort of nets was monitored annually for evidence of net deterioration and attrition.

**Results:**

After 12 months’ use, 97% of ICON Maxx-treated nets but only 67% of CTN passed the WHO efficacy threshold for insecticidal durability (> 80% mortality in cone or tunnel or 90% feeding inhibition in tunnel). After 24- and 36-months use, 67% and 26% of ICON Maxx treated nets met the cone criteria, respectively, and over 90% met the combined cone and tunnel criteria. Lambda-cyhalothrin content after 36 months was 17% (15.8 ± 4.3 mg/m^2^) of initial content. ICON Maxx nets were used year-round and washed approximately 4 times per year. In cross-sectional survey after 36 months the average number of holes was 20 and hole index was 740 cm^2^ per net. Cohort nets had fewer holes and smaller hole index than cross-sectional nets. However, only 15% (40/264) of cohort nets were not lost to follow-up or not worn out after 36 months.

**Conclusions:**

Because more than 80% of nets met the WHO efficacy criteria after 36 months use, ICON Maxx was granted WHO full recommendation. Cross-sectional and cohort surveys were complementary and gave a fuller understanding of net durability. To improve net usage and retention, stronger incentives and health messaging should be introduced in WHO LLIN longitudinal trials. Untreated polyester nets may be made long-lastingly insecticidal in Africa through simple household treatment using ICON Maxx pyrethroid-binder kits.

## Background

Long-lasting insecticidal nets (LLINs) are an important tool for malaria vector control. With LLIN technology, insecticidal efficacy is expected to be sustained against Anopheline mosquitoes for at least 3 years without need for further retreatment [1]. The proportion of the population with access to insecticide-treated nets (ITNs) and LLINs has increased markedly in sub-Saharan Africa over the past two decades. Manufacturers’ delivery data for 2004–2020 show that over 2.3 billion ITNs and LLINs were supplied globally in that period, of which 1.9 billion (86%) were supplied to sub-Saharan Africa [2]. By 2019, 68% of households in Africa had at least one ITN/LLIN, increasing from about 5% of households in 2000. The percentage of the population sleeping under ITNs or LLINs has increased from less than 2% in 2000 to 46% in 2019 [2]. Although highest numbers of LLINs are being delivered to sub-Saharan countries, 1 in 4 children live in households with no access to ITN or protection by indoor residual spraying [3]. To achieve one LLIN for every two household members, a ratio considered sufficient to achieve universal coverage [4], an estimated 200–300 million replacement nets are required each year to achieve and maintain universal access [3].

Owing to the insufficient number of LLINs being delivered through NMCPs and NGOs, many households use nets sourced locally through commercial and retail sectors. The majority of these are not LLINs and surveys show most have either never been treated or were treated only once on purchase [5–7]. These nets may be made from a variety of synthetic polymers or natural fibres. This emphasizes the need for long-lasting insecticide treatment kits that can be used to convert untreated nets into products that withstand repeated washing without need for annual retreatment. Such insecticide kits could be bundled together with untreated nets, sold from shops, and enable local net producers that lack LLIN manufacturing technology to produce a long-lasting ITN, which could address local LLIN shortages and contribute usefully to malaria control [1, 3].

Two brands of long-lasting treatment kit have been developed: KO-Tab 123 by Bayer Environmental Sciences [8] and ICON Maxx by Syngenta [9]. KO-Tab 123 treated nets remained insecticidal for 15–20 washes in WHO Phase II evaluation trials [8]. ICON Maxx is a twin-sachet treatment kit for treatment of individual family-sized polyester nets based on a slow-release capsule suspension formulation of lambda-cyhalothrin 10% CS and binding agent. Following bio-efficacy and wash-fastness studies in Phase I laboratory and Phase II experimental hut studies in Tanzania and Burkina Faso, WHO interim recommendation was granted to ICON Maxx [9, 10]. Further hut trials were run in Côte d’Ivoire [11]. Full WHO recommendation is only granted after demonstrating the candidate LLIN or long-lasting treatment kit meets specific efficacy criteria after 3 years of regular household use in large scale Phase III longitudinal trials [12, 13].

The main objective of the present study was to evaluate ICON Maxx treated nets in line with WHO guidelines for field testing of LLIN to determine insecticidal efficacy, wash fastness, acceptability, net integrity and net survivorship under East African household conditions over 3 years of use in comparison with a standard lambda-cyhalothrin 10% CS conventionally treated net without binder. Running in parallel with this Phase III longitudinal study presented here, a series of Phase I laboratory studies with ICON Maxx were run on a variety of polymer netting materials (polyethylene, polyester, nylon, cotton) to assess the treatment kit’s versatility on other types of netting and household substrates.

## Methods

### Study areas

**Fig. 1:**
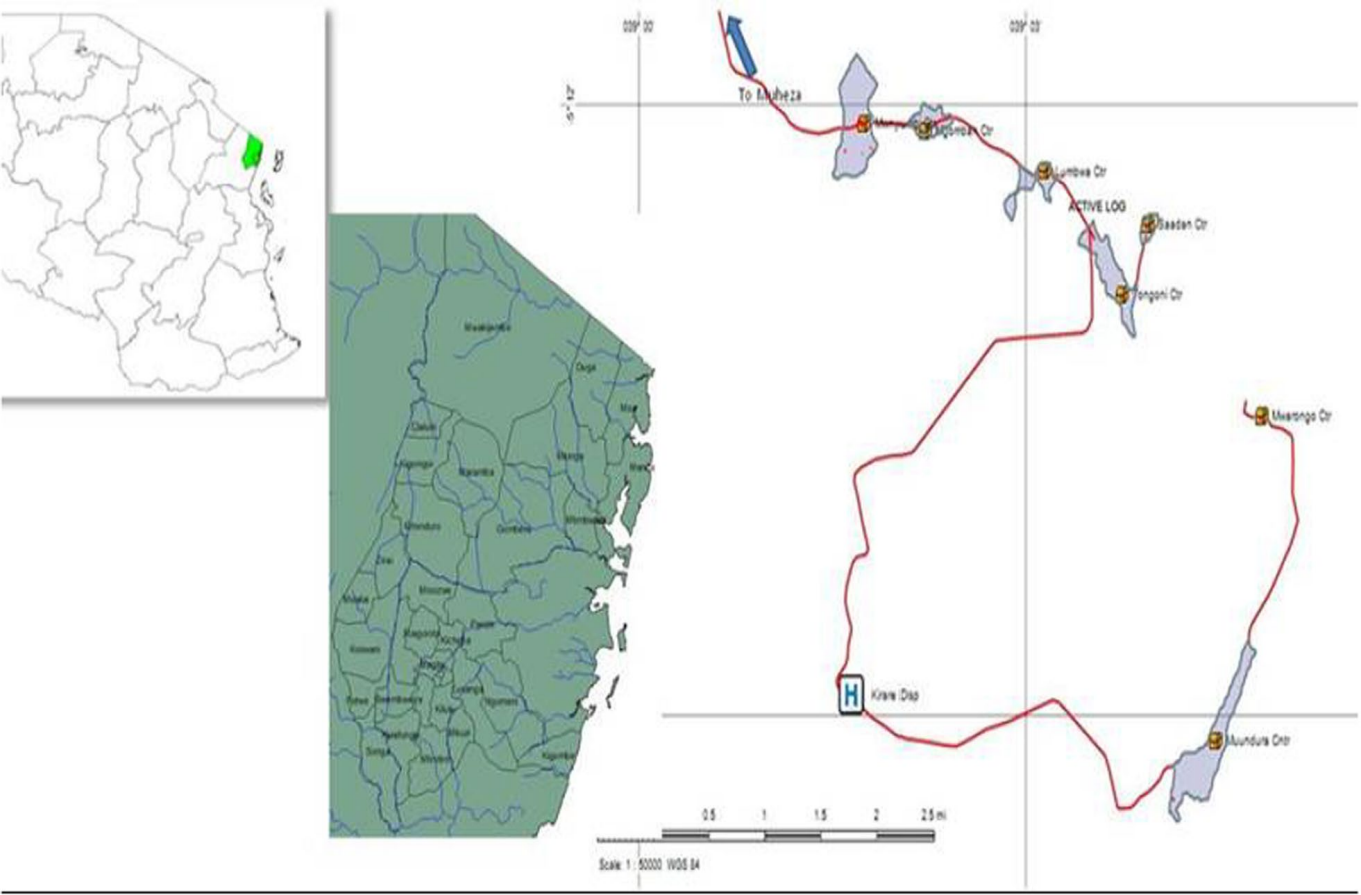
The Map of Tanzania (top left), Muheza district (middle) and the GPS-generated map of study area showing hamlet boundaries

The trial was conducted in the two coastal villages, Tongoni and Mwarongo, in Muheza and Tanga districts (5°10′ 0S; 38°46′ 0E) (Fig. 1). In the household demographic census survey, Tongoni village comprised 5 hamlets and 484 houses, Mwarongo village comprised 2 hamlets and 335 houses. The residents subsisted on maize, cassava and rice with some working on sisal plantations and others on orange plantations and animal husbandry. Annual rainfall was bimodal: short rains from October to December, long rains from March to June, ranging from 800–1400 mm per annum. Malaria transmission occurred most of the year, and there were two seasonal mosquito peaks during and after the long and short rainy seasons [14, 15]. During the rains *Anopheles gambiae *sensu stricto (*s.s.*) predominated, and in the dry season *Anopheles funestus* was more common. Malaria transmission is classified as holoendemic although some areas of the districts have a long history of ITN use [14, 16] and LLIN universal coverage campaigns took place in 2011 [7].

### Study design

The efficacy of ICON Maxx (Syngenta, Switzerland) treated polyester nets and nets conventionally treated with lambda-cyhalothrin CS (Iconet CS, Syngenta, Switzerland) at WHO recommended dosages were compared under field conditions in a two-arm household randomized trial with the household as the unit of randomization and mosquito nets as the unit of observation. The conventionally treated nets (CTN) were studied for one year after which all households with the Iconet-treated CTN were replaced with ICON Maxx treated nets [12]. The efficacy of ICON Maxx treated nets were monitored for up to 3 years of continuous use. It should be noted that the study coincided with the publishing of the 2013 revised WHO Guidelines for Laboratory and Field Testing of LLIN which recommended that a candidate LLIN or long-lasting treatment kit should be field evaluated with reference to an existing WHO-recommended LLIN rather than a CTN [13]. The WHO made an exception for Phase III trials already in progress.

Seven cross sectional surveys were undertaken, the first was carried out 1 month after net distribution, subsequent surveys took place every 6 months. A random sample of 30–50 households from each arm was selected every 6 months from the master list of participating households and subjected to physical integrity inspection, cone bioassay and tunnel tests. In year 1, both arms were surveyed, in years 2 and 3 only the ICON Maxx arm was surveyed.

Additional cohorts of 250 nets from a randomly selected 100 households from each arm were followed up annually for 3 years in the case of ICON Maxx and for 1 year in the case of Iconet CTN for assessment of survivorship and attrition in accordance with the revised WHO guideline [13].

### Treatment of nets with ICON Maxx and Iconet

A total of 2500 polyester nets were treated individually with long-lasting ICON Maxx (Syngenta, Switzerland) from twin sachet packs, containing 7.3 ml of lambda-cyhalothrin 10% CS and 7.7 ml of binding agent. Nets were available in three widths (180, 150 and 120 cm) according to family needs and sleeping arrangements and were all 180 cm long and 150 cm high. ICON Maxx solution was applied by hand individually in basins according to the manufacturer’s instructions using the appropriate volume of solution for each size of net to give a target dose of 62 mg AI/m^2^. A safety assessment concluded that no unacceptable exposures were found in the preparation, maintenance and use of the nets over the prescribed dose range of 50 (for family net) to 83 (for single net) mg AI/m^2^ [9].

A further 1250 conventionally treated polyester nets were treated with lambda-cyhalothrin 10% CS sachet (Iconet 10% CS, Syngenta Switzerland) without binder to a recommended target dose of 15 mg AI/m^2^ [8]. Treatment of nets was carried out by a trained team of field workers under supervision of the principal investigator. During treatment field workers wore personal protective clothing including gloves and masks. Treatment was done outdoors in the open air and nets were dried on plastic sheeting under shade. Nets were turned over periodically until dripping stopped and then hung-over washing lines to complete the drying.

### Household randomization and net distribution

Houses were numbered during a village and household census. After the census, the ICON Maxx nets and CTNs were distributed to each household door-to-door in late June and early July 2011. A total of 1250 ICON Maxx nets and 1250 CTNs were distributed. A further 1250 ICON Maxx nets were held back to replace the 1250 CTNs at the end of the first year. The distribution was stratified by household so that each net type was present in each hamlet in a 1:1 ratio. Individual households received either ICON Maxx or Iconet CTNs rather than a mix of types. A unique code number was written on each net using a permanent marker. Sufficient nets were distributed to cover all sleeping spaces in each house. Householders were informed about the need for reporting adverse effects during net use and advised on proper use and maintenance. Assistance in hanging nets over the sleeping places was given where needed.

### Household surveys and net integrity (cross-sectional surveys)

During cross-sectional household surveys nets were sampled from each treatment arm at six-monthly intervals. Both ICON Maxx and CTNs were randomly sampled at 6 and 12 months after distribution; thereafter, only ICON Maxx nets were sampled after 18, 24, 30 and 36 months of use. The 30 households sampled per survey (50 in the final 36-month survey) were selected at random using the household ID master list, one net from each household was sampled, the selected household received a replacement ICON Maxx net and then removed from the study. At the time of each cross-sectional survey and net collection, a household questionnaire was applied to assess net use, acceptability, washing practices and any adverse effects.

Net integrity surveys were carried out every 6 months for 36 months in the randomly selected ICON Maxx houses and after 6 and 12 months in the randomly selected CTN houses. Each selected net was hung over a wooden frame and scored for size and distribution of holes, repairs (stitches, knots and patches) and open/failed seams. Assessment of cleanliness was done concurrently and nets categorized according to their degree of dirtiness. Hole sizes were categorized as size 1—smaller than a thumb, size 2—larger than a thumb but smaller than a fist, size 3—larger than a fist but smaller than a head, size 4—larger than a head. Hole index was estimated using the method defined by the WHO, which assumes that the hole size equates to the mid-point of the range for each hole size category [13]. The estimate of hole area gives a slightly more conservative value when compared to the hole index [17].

### Net attrition and functional survivorship (cohort surveys)

Two cohorts of 100 houses from ICON Maxx and Iconet CTN arm were selected, censused and nets checked at the end of each study year (after 12, 24 and 36 months) for condition, attrition and functional survivorship. Study nets were recorded as present, discarded due to damage (wear and tear, rodent and burn holes) or lost to follow up. Households where the inhabitants were absent or where nets were recorded as given away, used elsewhere, stolen or lost were not included in the estimates of functional survivorship. Functional net survivorship was based on damage only and did not include in numerator or denominator nets lost to follow up due to family movement, theft, gifted or sold as these nets might still be functional.

### Chemical analysis

From each of the ICON Maxx and CTN sampled at baseline and surveyed at 12, 24, 30 and 36 months, five additional pieces of netting (30 cm × 30 cm) were cut for chemical analysis from each of the five panels of each net. The piece closest to the mattress line was excluded as per WHO guidelines [12]. All pieces were sent to the WHO-collaborating at the Centre Wallon de Recherches Agronomiques (CRA-W) for chemical analysis. The net pieces from each net were pooled, cut into small pieces and homogenized, and lambda-cyhalothrin was extracted and quantified using gas chromatography CIPAC 463/LN/M/3 [18].

### Bio-efficacy and residual activity of nets

From the 30 to 50 ICON Max nets and CTNs sampled every 6 months, 5 netting pieces measuring 25 cm × 25 cm were cut from the five panels of each net in accordance with WHO guidelines [12]. Cone bioassay tests were carried out on the netting pieces at the NIMR Amani Centre using 2–5 day old, unfed, female *An. gambiae s.s.* (Kisumu). Twenty mosquitoes were exposed in 4 replicates of 5 mosquitoes to pieces from positions 2–5 of each net (total of 80 mosquitoes per net) for 3 min in WHO plastic cones; the piece where abrasion was greatest (tucked under the mattress) was excluded as recommended by WHO [12]. After exposure the mosquitoes were held in paper cups at 26 °C and 80% relative humidity and given access to 10% glucose solution. Knockdown was recorded 1 h after exposure and mortality after 24 h. When knockdown was < 95% and mortality was < 80%, the net was subjected to tunnel testing [12]. The net piece closest to average mortality of the net was tested in the tunnel. Any net meeting the cone criteria of ≥ 80% mortality or ≥ 95% knockdown or tunnel test criteria of ≥ 80% mortality or ≥ 90% blood-feeding inhibition was considered to have met the required threshold.

### Data analysis

Data were double-entered into Microsoft Access and analysed in STATA version 10. Comparison of chemical content between net types over time was analysed using analysis of variance and t-tests. Wilcoxon rank sum test was used to analyse continuous data that was not normally distributed. Logistic regression was used to analyse the association between percentage knockdown and 24-h mortality with washing, net usage and insecticide content. Chi-squared test for trend was used to analyse net efficacy over successive surveys. Poisson regression was used to the test for association between hole index and time, number of washes and net usage.

### Ethics, consent and permission

Ethical clearance was obtained from the ethics committees of the NIMR Tanzania (Ref: NIMR/HQ/R.8a/Vol X/86) and London School of Hygiene and Tropical Medicine. Written informed consent was obtained from all household heads of participating families.

## Results

### Household surveys

A total of 705 households were identified in the baseline survey; 70% had mud walls and 75% palm thatched roofs. Other roofing materials included corrugated iron, some walls were made of brick. The mean ages of household heads in ICON Maxx and Iconet households were 43 and 52 years respectively. Most householders were farmers (33% ICON Maxx, 55% Iconet); the remainder were employed as fishermen, teachers, nurses, students or unemployed. The majority (65% ICON Maxx, 78% Iconet) had received 7 years of primary school education, 7% and 20% had received secondary education and others had not gone to school at all.

Both the ICON Maxx nets and Iconet CTNs were well-accepted by the communities. Reported net use was 100%. Respondents indicated using their nets year-round and every night. Almost 90% of surveyed nets were found hanging above beds and 10% were observed suspended over floor mattresses. 72% and 78% of sampled populations stated their reason for using nets was to protect themselves from mosquito biting while 13% and 28% stated for protection from malaria**.** The frequency of net washing was found not to differ between ICON Maxx net surveys. Estimated washing frequency was 4 times per year (Table 1).

**Table 1:** Washing frequency and net appearance

Survey (month)	IconMaxx						lambda-cyhalothrin CTN					
	No. nets	Mean (SD) no. of washes^a^	General aspect of nets %				No. nets	Mean (SD) no. of washes^a^	General aspect of nets %			
			Clean	Slightly dirty	Dirty	Very Dirty			Clean	Slightly dirty	Dirty	Very Dirty
Cross section survey												
0	30	0	30	0	0	0	30	0	30	0	0	0
6	30	3 (2.0)	7	21	69	3	30	2 (1.7)	18	26	52	4
12	30	1 (0.3)	45	0	39	16	30	1 (0.4)	36	3	36	25
24	30	4 (1.5)	13	13	55	19	–	–	–	–	–	–
36	50	4 (2.6)	16	6	56	22	–	–	–	–	–	–
Cohort survey												
0	264	0	100	0	0	0	266	0	266	0	0	0
12	98	6 (2.0)	29	7	54	10	113	6 (2.0)	36	5	52	7
24	46	6 (3.2)	28	14	46	12	–	–	–	–	–	–
36	40	7 (3.5)	26.5	26.5	15	32	–	–	–	–	–	–

^a^Mean number of washes per year

All respondents in all surveys reported washing their nets in cold water. Nobody reported rubbing nets against rocks or on washing stones. Nets were pre-soaked by 18–40% of respondents; soaking times ranged from 10 min to 4 h. Nets were reported washed using commercial bar soap (30–85%), detergent powder (14–50%) or both (14–32%). Most nets (92–98%) were rinsed after washing and most (92–98%) were dried outdoors.

Despite householders reporting high frequency of ICON Maxx net washing, it was observed that only 45% and 16% were scored as clean at 12 months and 36 months respectively and 19% and 22% were scored as very dirty at 24 months and 36 months respectively. There was no association between the alpha-cypermethrin content remaining on the nets at 36 months and the reported number of washes (F_1,48_ = 1.2, P = 0.30). Nor was there any association between the reported number of washes over 36 months and the proportion of nets failing the cone bioassay criterion (F_1, 48_ = 0.3, P = 0.85).

### Physical integrity of nets in cross-sectional surveys

The same brand of 100-denier nets was used in ICON Maxx and Iconet CTN arms. In the baseline survey there were no holes or open seams on any of the sampled nets in the ICON Maxx or CTN arms. After 6 months, approximately half of the nets (52% of ICON Maxx nets, 56% of Iconet CTNs) had at least one hole (these were mainly of the smallest size category) (Table 3); the mean number of holes per net was 4.3 for ICON Maxx nets and 5.5 for CTN (Table 2). The number of holes increased between 6 and 12 months to a mean of 9 for ICON Maxx arm and 7 for Iconet arm; the majority of holes were always found in the lower part of the panels (Table 3). Comparison of physical integrity between ICON Maxx and CTN nets confirmed there was no difference between either arm at 12 months when the CTNs were disused (Tables 2, 3). After 24 months, while most ICON Maxx nets (84%) had a least one hole, these remained of the smallest hole category (Tables 2, 3); the mean number of holes per net had increased to 14.5 (Tables 2, 3), the mean hole index (HI) was 589, the median HI was 197 (IQR = 352) and the geometric mean number was 71 (Table 4). After 36 months, most hole indices had increased: 82% of nets were holed (Tables 2, 3), the majority were still size 1 (59%), the mean HI was 740, the median HI was 417 (IQR = 615) and the geometric mean number was 59 (Table 4).

**Table 2:** Physical condition of IconMaxx and CTN by survey round holes by size category

Survey (month)	ICON Maxx						lambda-cyhalothrin CTN					
	No. of nets	Mean no. of holes per net	%Percentage of holes per size				No. of nets	Mean no. of holes per net	%Percentage of holes per size			
			1	2	3	4			1	2	3	4
Cross section survey												
0	30	0	0	0	0	–	30	0	0	0	0	–
6	30	4	58	29	13	–	30	5	44	26	30	–
12	30	9	72	21	7	–	30	7	57	30	13	–
24	30	14	53	36	11	–	–	–	–	–	–	–
36	50	20	59	31	8	2	–	–	–	–	–	–
Cohort survey												
0	264	0	0	0	0	–	266	0	0	0	0	–
12	98	5	54	34	12	–	113	3	56	32	12	–
24	46	11	66	25	9	–	–	–	–	–	–	
36	40	15	41	50	7	2	–	–	–	–	–	–

**Table 3:** Physical condition of IconMaxx and CTN by survey round holes by distribution

Survey (month)	ICON® MAXX LLIN							lambda-cyhalothrin CTN						
	No. of nets	% nets w/at least 1 hole	% holes by distribution^a^			Mean no. of open seams	% nets with any repairs	No. of nets	% nets w/at least 1 hole	% holes by distribution^a^			Mean no. of open seams	% nets with any repairs
			Lower	Upper	Roof					Lower	Upper	Roof		
Cross section survey														
0	30	0	0	0	0	0	0	30	0	0	0	0	0	0
6	30	52	82	12	6	0.3	20	30	56	81	14	5	0.6	0
12	30	74	89	3	8	0.6	30	30	72	90	9	1	0.5	0
24	30	84	67	22	11	1.2	13	–	-	-	-	-	-	-
36	50	82	74	11	15	0.9	32	-	-	-	-	-	-	-
Cohort survey														
0	264	0	0	0	0	0	0	266	0	0	0	0	0	0
12	98	48	86	5	9	0.3	6.7	113	42	84	11	5	0.3	2.6
24	46	67	77	12	1	0.6	5	-	-	-	-	-	-	-
36	40	85	67	21	12	1.4	20	-	-	-	-	-	-	-

^a^Location of holes: lower, lower half of side panels; upper, upper half of side panels; roof, top panel

**Table 4:** Physical integrity–comparison of estimates of the average hole index, hole area between cross-sectional and cohort surveys for ICON Maxx and Lambda-cyhalothrin CTN

Survey type	Survey (month)	ICON Maxx LLIN							lambda-cyhalothrin CTN						
		No. of nets	Hole index			Hole area cm^2^			No. of nets	Hole index			Hole area cm^2^		
			Mean (SD)	Median (IQR)	GM^a^	Mean (SD)	Median (IQR)	GM^a^		Mean (SD)	Median (IQR)	GM^a^	Mean (SD)	Median (IQR)	GM^a^
Cross section	0	30	0	0	0	0	0	–	30	0	0	0	0	0	0
Cohort		264	0	0	0	0	0	–	266	0	0	0	0	0	0
Cross section	12	30	513 (2048)	13 (162)	17	244 (865)	15 (162)	13	30	214 (425)	24 (265)	19	126 (206)	29 (163)	16
Cohort		98	166 (326)	0 (196)	8	101 (183)	0 (138)	7	113	114 (261)	0 (57)	6	67 (139)	0 (65)	5
Cross section	24	30	589 (1017)	197 (352)	71	373 (767)	86 (352)	49	–	–	–	–	–	–	–
Cohort		46	271 (435)	27 (415)	23	164 (247)	31 (204)	19	–	–	–	–	–	–	–
Cross section	36	30	740 (986)	417 (615)	59	483 (632)	226 (615)	101	–	–	–	–	–	–	–
Cohort		40	535 (614)	300 (876)	133	382 (393)	271 (738)	80	–	–	–	–	–	–	–

The age of nets (number of months of use) was positively associated with net HI (F_1,345_ = 9.31, P = 0.002). There was no association between the reported number of washes per net and net HI (R^2^ = 0.015, P < 0.4027), suggesting that frequency of reported washing was not associated with net durability. Nor did any differences in type of washing agent used have any association with net durability (F = 0.03, P < 0.969).

While the mean number of holes per net, the hole index and hole area showed an increasing trend between 0 and 36 months, no more than 13% of holes were ever greater than size 2 (Tables 2, 3, 4).

### Net efficacy through bioassay

**Fig. 2:**
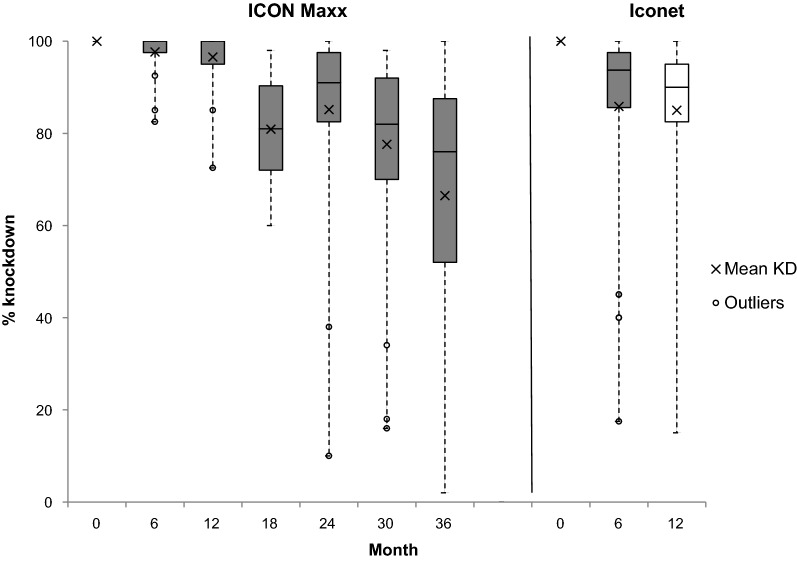
Median (IQR) and mean percentage *An. gambiae s.s.* (Kisumu) knockdown 1 h post-exposure to ICON Maxx and CTN pieces in cone bioassays

**Fig. 3:**
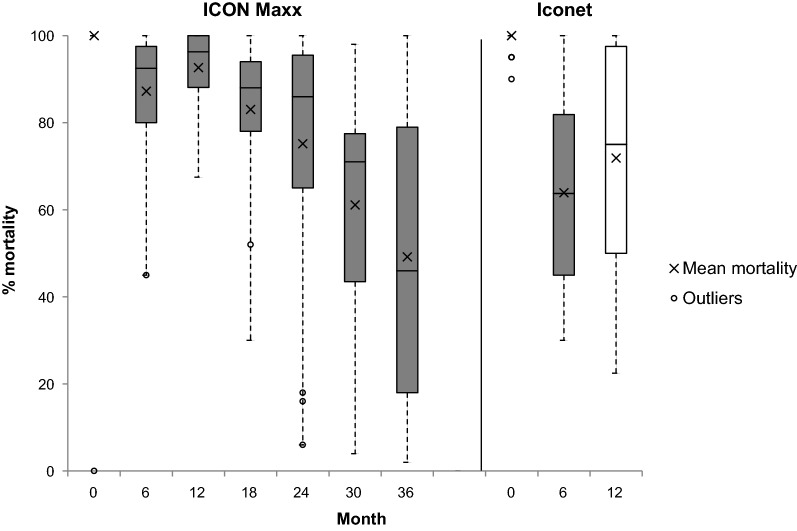
Median (IQR) and mean percentage *An. gambiae s.s.* (Kisumu) mortality 24 h post-exposure to IconMaxx and CTN pieces in cone bioassays

**Fig. 4:**
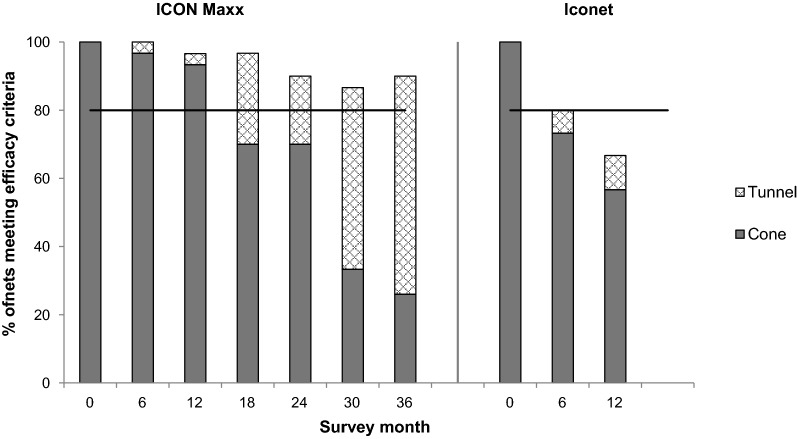
Percentage ICON Maxx & CTN meeting WHO efficacy criteria (solid bar = cone test, hatched bar = tunnel test) by survey round. The horizontal line represents the acceptability cut-off for WHOPES full approval of the LN

Baseline cone bioassay tests on ICON Maxx and CTN (Iconet) after treatment but before distribution resulted in 100% knockdown and 100% 24-h mortality on all pieces tested (Figs. 2 & 3). After six months of use the mean percentage mortality (± 95% CI) was significantly greater on ICON Maxx than on CTN (87.2%, CI 82–92 vs 63.9%, CI 56–87, p < 0.0001); similarly, mean percentage knockdown on ICON Maxx was significantly greater than that on the CTN (97.7%, CI 96–99 vs 86%, CI 78–94, p < 0.004) (Figs. 2 & 3). The survey after 12 months of use continued to show differences between ICON Maxx and CTN treatments in mean percentage mortality (93%, CI 86–96, vs 72%, CI 62–81, p < 0.0004) and mean percentage knockdown (97%, CI 94–99, vs 85%, CI 78–91, p < 0.0005) (Figs. 2 & 3). With respect to pass rate, 1 ICON Maxx and 8 CTN failed the cone after 6 months of use (Fig. 4). Some nets that failed the cone test subsequently passed the tunnel test criteria (1 ICON Maxx and 2/8 CTN), producing combined test pass rates of 100% for ICON Maxx and 80% (24/30) for CTN at 6 months (Fig. 4, Table 5). After 12 months of use, whilst the combined test pass rates remained high for ICON Maxx nets at 96.7% (29/30), it was much lower for the CTN at 66.7% (20/30) (Fig. 4, Table 5). Bioassays on the CTN were discontinued forthwith. Subsequent surveys focused on the ICON Maxx nets. After 18 months fewer ICON Maxx nets passed the cone test (70%, 21/30) but combined cone and tunnel testing produced an overall pass rate of 96.7% (29/30) similar to the pass rate at 12 months (Fig. 4, Table 5). After 24 months the combined pass rate remained high at 90% (27/30). After 30 months, although fewer nets passed the cone test criteria (33%, 10/30), the majority of nets that failed the cone tests achieved the tunnel test criterion (80%, 16/20) producing an overall pass rate of 86.7% (26/30). At 36 months, although only 26% (13/50) of ICON Maxx nets passed the cone criterion, 86.5% (32/37) of nets that failed the cone achieved the tunnel test criterion producing an overall pass rate of 90% (45/50) (Fig. 4). The incremental decrease in pass rate over the full 36 months was small but significant (χ^2^_trend_ = 11, P = 0.001). The incremental decrease in cone test pass rate was highly significant over the full 36 months (χ^2^_trend_ = 34, P = 0.001).

**Table 5:** Percentage of ICON Maxx and Iconet CTN meeting WHO efficacy criteria by survey round

Survey (month)	ICON Maxx			lambda-cyhalothrin CTN		
	Cone bioassays	Tunnel tests	Cone and tunnel tests combined	Cone bioassays	Tunnel tests	Cone and tunnel tests combined
0	100 (30/30)	–	100 (30/30)	100 (30/30)	–	100 (30/30)
6	96.7 (29/30)	100 (1/1)	100 (30/30)	73.3 (22/30)	25 (2/8)	80 (24/30)
12	93.3 (28/30)	50.0 (1/2)	96.7 (29/30)	56.7 (17/30)	23 (3/13)	66.7 (20/30)
18	70.0 (21/30)	88.9 (8/9)	96.7 (29/30)	–	–	–
24	70.0 (21/30)	66.7 (6/9)	90.0 (27/30)	–	–	–
30	33.3 (10/30)	80.0 (16/20)	86.7 (26/30)	–	–	–
36	26.0 (13/50)	37.0 (32/37)	90.0 (45/50)	–	–	–

### Analysis of chemical content and insecticide retention (Fig. 5 and Table 6)

**Fig. 5:**
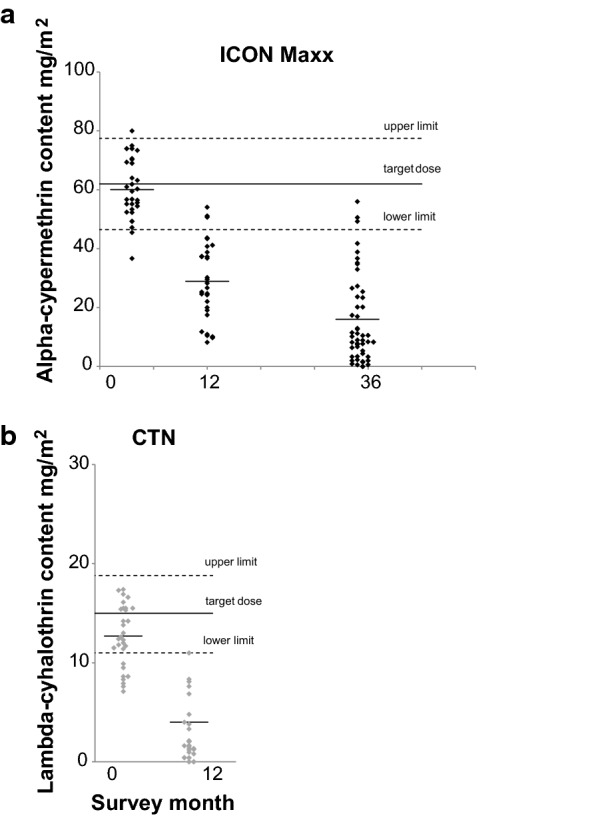
**a** Lambda-cyhalothrin content (mg AI/m^2^) on individual IconMaxx nets samples at baseline after 12 and after 36 months of field usage for IconMaxx nets. Mean concentrations for each time point are indicated by the thin horizontal lines. The target dose and upper and lower limits are for lambda-cyhalothrin content at baseline indicated as solid and dashed lines.** b** Lambda-cyhalothrin content (mg AI/m^2^) on individual CTN samples at baseline after 12-monthly intervals of field use for Iconet. Mean concentrations for each time point are indicated by the thin horizontal lines. The target dose and upper and lower limits are for lambda-cyhalothrin content at baseline indicated as solid and dashed lines

**Table 6:** Lambda-cyhalothrin content (mg AI/m^2^) on ICON Maxx nets and CTN at baseline and after field use

Survey (month)	ICON Maxx-treated nets			Iconet CTNs		
	N	Mean	%AI loss	N	Mean	%AI loss
		mg AI/m^2^			mg AI/m^2^	
0	30	60.1 (56.3–63.9)	–	30	12.7 (11.5–13.9)	–
12	30	28.9 (23.9–33.9)	52%	30	4 (0.9–7.1)	68%
36	50	15.8 (11.5–20.1)	74%			

At baseline, the mean lambda-cyhalothrin content of ICON Maxx treated nets was 60.1 mg AI/m^2^ (Fig. 5a); this was very close to the target of 62 mg AI/m^2^. At baseline the mean lambda-cyhalothrin content among CTN was 12.7 mg AI/m^2^, well within the acceptable limits of the target dose of 15 mg AI/m^2^ (Fig. 5b). After 12 months of household use the lambda-cyhalothrin content of ICON Maxx had decreased to 28.9 mg AI/m^2^ corresponding to 52% loss of the baseline AI content (Fig. 5a); the content of CTN decreased to 4 mg AI/m^2^ after 12 months corresponding to 68% of baseline AI content (Fig. 5b). After 36 months the mean lambda-cyhalothrin content on the surveyed ICON Maxx nets was 15.8 mg AI/m^2^ (n = 50, RSD = 93.4%) corresponding to 73.7% loss of the original content (Table 6).

The mean lambda-cyhalothrin concentration on ICON Maxx treated nets that passed the cone bioassay criteria at 36 months was 30.4 (21.4–39.4) mg AI/m^2^, while content on nets that failed the cone criteria was 10.7 (7.5–13.9) mg AI/m^2^; the difference in AI content between failing nets and those passing cone test criteria was significant (F_1,48_ = 26.1, P = 0.0001).

The mean concentration on nets that failed the tunnel test criteria was 5.7 mg/m^2^ and on those that passed was 16.9 mg/m^2^; the difference in AI content between pass and fail was significant (t = 2.5, P = 0.009).

### Adverse effects among staff treating nets and families using nets

Three attendants were responsible for treating nets at the start of the project at a rate of 60 nets per person per day for 2 weeks. All attendants who treated the nets reported sneezing and facial itching, and one reported fever. The adverse effects were more common after treating with Iconet (CTN) than after treating with ICON Maxx, even though the treatment dose was higher for ICON Maxx. One of the attendants regularly reported irritation to facial skin (paraesthesia). The effect took about 3 h to subside on each occasion. The discomfort was not so severe that the individual took time off work. All proper precautions were taken while treating the nets including wearing of masks and gloves.

Of the 60 households included in the first week and first month post-treatment surveys, only a small proportion reported experiencing any adverse effects and only during the first few days of net use. Similar proportions of Iconet CTN users (10%) and ICON Maxx net users (6%) reported these effects which included bad odour, sneezing, skin itching, nasal discharge and facial itching. The effects were transient and did not deter users from continuing to use the nets. No adverse effects were reported after one month of use. During the 6 months survey the interviewees reported that all symptoms stopped after the net had been washed once. At no stage did any of the adverse events require medical attention.

### Net attrition and survivorship rate

**Fig. 6:**
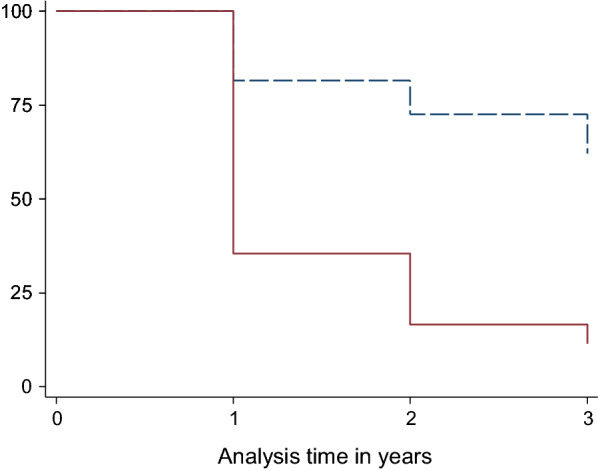
Kaplan–Meier Nets Survival Curve; Hatched line = Net discarded due to damage, Solid line = Net discarded due to damage or lost to follow-up)

Net survivorship due to loss of integrity (accumulated holes) caused by physical deterioration or damage fell from 100 to 78% after 12 months, to 70% after 24 months and to 68% after 36 months (Fig. 6 & Table 2b). A very high proportion of nets distributed (72%, 189/264) were lost not due to loss of integrity but to more mundane reasons such as moving house to outside the study area, hut collapse, or nets being given away during the 36 months. For most of the nets that were lost to follow up this occurred between 0 and 24 months.

**Fig. 7:**
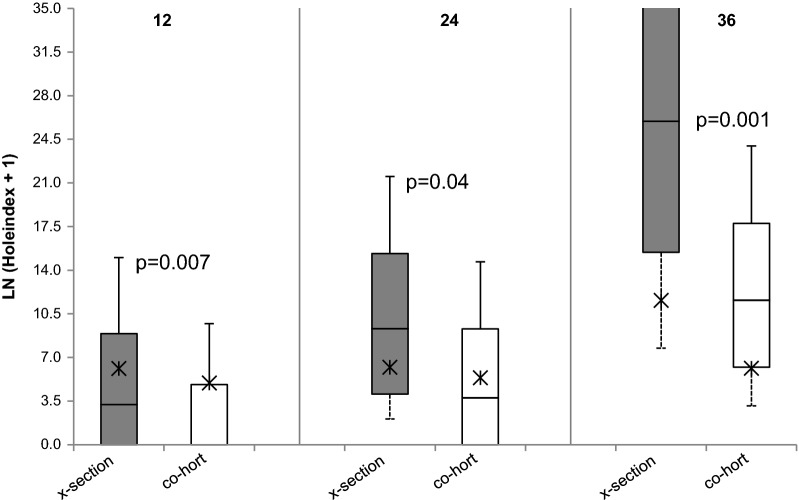
Comparison of median (IQR) and mean ln (hole index + 1) of the ICON Maxx nets between cohort and cross section surveys at 12-, 24- and 36-month’s survey points

In the comparison of physical integrity of nets between the cohort-longitudinal surveys and cross-sectional surveys, the majority of holes in the cohort surveys were found in the lower part of the panels (Table 3 & Fig. 7). In all three cohort surveys the hole indices were significantly lesser (Z = 2.46; P = 0.014 for 12th month, Z = 2.6; P = 0.009 for 24th month and Z = 6.14; P = 0.001 for 36^th^ month surveys) than in the cross-sectional surveys after the corresponding periods of use (Table 3 & Fig. 7). This may reflect cohort members’ self-awareness that they were being monitored more closely than other recipients of ICON Maxx treated nets.

Attrition and physical integrity of the ICON Maxx-treated nets were monitored as recommended in the WHO LLIN testing guidelines. As a home-treatment kit, it should be noted that net integrity and hole index were never part of the product claim of ICON Maxx. Nevertheless, it was important to compare net integrity and bio-efficacy as part of the evaluation of ICON Maxx and to correlate the formulation performance with polyester net condition, as well as consider other types of material that ICON Maxx might be called upon to treat.

## Discussion

### Testing, bioefficacy and recommendation

The present study evaluated the efficacy of ICON Maxx treated nets for up to 36 months of household use using the standard WHO cone bioassay criteria of knockdown and mortality and the tunnel test criteria of mortality and blood feeding inhibition. According to WHO testing guidelines [13] a candidate LLIN or long-lasting treatment kit is deemed to meet the requisite threshold for WHO recommendation if, at the end of 3 years use, at least 80% of the sampled nets retain bio-efficacy using any of the WHO bioassay criteria [13].

Applying the WHO criteria, ICON Maxx failed to achieve the 80% threshold at the 36-month sampling point on the basis of cone bioassay alone. On inclusion of the tunnel test data, more than 90% of ICON Maxx treated nets met the efficacy criteria for the combined cone test and tunnel test. While the bio-efficacy of ICON Maxx treated nets remained high throughout the 36-month study period, the comparator arm of Iconet CTN was dropped from the trial after 12 months because at that sampling point it fell short of the required 80% pass rate and was considered unethical to continue. At that point, the CTN households were provided with ICON Maxx sachets and instruction leaflets and verbal guidance given.

Examining the bioassay methods individually, the ICON Maxx nets met the WHO cone efficacy criteria for the first 12 months. In the 2nd year fewer than 80% of nets met the WHO cone criteria, and the tunnel test played an increasingly important role. By the 36-month survey point, 74% of sampled ICON Maxx nets were failing the cone test and the tunnel testing was required to achieve the 80% pass rate. Contrast the long-lasting treatment kit results with other net products tested in Phase III trials at NIMR Muheza, such as Interceptor LN [17], a factory produced long lasting insecticidal net. After 36 months, a much higher proportion of Interceptor LN, 77% (23/30), met the cone criteria and only a few cone-failures needed to go forward to tunnel testing criteria to take Interceptor LN over the line. By contrast, with the ICON Maxx sampled at 36 months only 26% (13/50) met the cone criteria and a further 86% (32/37) had to go forward to tunnel testing to take ICON Maxx over the line. Why the difference? Was it differences in binder constituents between ICON Maxx and Interceptor LN or was it differences in binding process: factory versus community? The two-Phase III studies were comparable: both were done at the NIMR Amani Centre by the same scientific group in consecutive years. But because the two alphacyano-pyrethroids (alpha-cypermethrin in Interceptor LN and lamba-cyhalothrin in ICON Maxx) and the loading dosages (200 mg/m^2^ in Interceptor LN and 62 mg/m^2^ in ICON Maxx) were not the same in the two products, the differences in efficacy cannot be attributed with any certainty to differences in binder technology (factory machine versus field hand treatment) or binder composition. However, the proportions of loading concentration lost over 12 to 36 months in the field were in fact remarkably similar: 49% in Interceptor and 48% in ICON Maxx after 12 months, and 82% in Interceptor and 74% in ICON Maxx after 36 months [19, 20]. It may not be a question of quality of binding agent or within-net heterogeneity between factory treatment versus community treatment but simply a question of different pyrethroid and the higher concentration of AI applied under factory conditions. Notwithstanding the true reason, the Tanzanian ICON Maxx samples did meet the bio-efficacy criteria, the overall study was reviewed by the WHO and ICON Maxx was given full recommendation as a long-lasting net treatment for up to 3 years of use.

Besides Tanzania, the only other significant Phase III trial of ICON Maxx sponsored by WHO was conducted in India in Odisha state [19, 21]. Relatively poorer performance of ICON Maxx nets was reported, with only 59% of the ICON Maxx nets meeting efficacy criteria with the combined cone test or tunnel test after 36 months. While 80% passed the combined cone-tunnel after 30 months, a much smaller proportion of the tested *Anopheles stephensi* responded adequately (41% mortality in the tunnel after 36 months and only 17% of nets passing the tunnel criteria) as compared to the 81% mortality in the tunnel with *An. gambiae* after 36 months in Tanzania and 86% of nets passing the tunnel criteria. Another difference notably at odds with the Tanzanian trial was high AI retention of lambda-cyhalothrin in ICON Maxx nets after 36 months (34 mg/m^2^ or 55% of loading content), potentially due to the shorter season of net use in India each year, as compared to 16 mg/m^2^ or 26% of loading content retained after 36 months and the much longer period of use in Tanzania. While the tunnel test is a highly realistic bioassay and simulator of experimental hut trials, there are clearly outstanding questions about vector responsiveness to bait in the tunnel and comparative performance of the different mosquito species that need to be resolved.

Beyond ICON Maxx, several brands of standard LLIN have achieved full WHO recommendation. Among the polyester LLIN they include PermaNet 2.0 LN and Interceptor LN [9, 22]. Among the polyethylene LLIN they include Olyset LN and Duranet LN [20, 23]. A further four brands have obtained WHO full recommendation on the basis of equivalence to the aforementioned brands, and a further nine have obtained WHO interim recommendation after demonstrating bio-efficacy in Phase II experimental hut trials [24]. The main purpose of ICON Maxx, in having achieved full recommendation, is not to rival these brands of LLIN (which it could do) but to facilitate the treating of untreated nets, acquired commercially, in community or home, or for re-treating currently used nets between universal coverage campaigns if the gap is proving too long.

As mentioned, net integrity and hole index is not part of the product claim. Nevertheless, as part of the evaluation it was important to compare net integrity and bio-efficacy with what owners’ report. This is particularly important when, as a cultural norm, the net recipients may not be allowed free access to the home to inspect the nets in situ. For example, nets became quite dirty within a year, and stayed dirty despite owners claiming to wash them every few months. There was no association between the reported number of washes at 36 months and the proportion of nets passing the cone bioassay criteria or with how much alpha-cypermethrin remained on the net. There was no association between the reported number of washes per net and net integrity (hole index). The only correlation observed was between the reported frequency of net use and net integrity or hole index. Clearly the hole index is a key indicator to retain with longitudinal studies of this kind. The positive association between bioassay outcome and AI content was reassuring to observe, as was the association between hole index and loss of bioassay efficacy or loss of AI content over time. Net cleanliness and reported number of washes were not reliable. The respondents may for personal reasons feel bound to give responses which they think will encourage the interviewer even when this is not the aim; for example, they may wish to report relatively higher number of washes with the purpose of showing that the net is being well cared for. The study did also highlight that washing was not the sole cause of insecticide removal; physical abrasion and friction during daily use was obviously a major factor too.

### Net integrity and durability

While many LLIN brands may achieve the requisite WHO bio-efficacy, few LLINs can withstand the abrasion and wear and tear of 3 years of field use. Survival will depend on the local environment and conditions of use. After 36 months, the majority of treated nets were lost or damaged: 18% were without holes and most were dirty. The condition of the polyester nets was consistent with that of the factory treated polyester LLIN previously evaluated in the same district where only 17% were without holes and 70% were dirty [17]. Both cross-sectional and cohort longitudinal surveys were used in monitoring of net integrity and the accumulation of holes. Nets sampled during cross-sectional surveys were always in worse condition as compared to cohort nets surveyed at the same time point. One possible explanation is that cohort households were better informed from the outset of their involvement in the longitudinal study than were cross sectional households. With the insight that their nets would be periodically inspected, this might have influenced cohort households to look after their nets better than the cross-sectional households did. Another possible reason was cultural. According to the norms of the society living in the study area, most householders were not willing to allow access to field workers to their home to randomly select the net for cross sectional survey. In most cases it was the householders who sampled the net to give to the field workers. Their awareness that the sampled net would be replaced by a new one could influence them to select the net of worse condition as a way of discarding a net nearer its end of life. This might have led to net sampling bias during cross sectional surveys leading to samples of worse condition compared to cohort nets.

As shown by the present trial, nets are lost to follow-up for a variety of reasons apart from deterioration or attrition. This might include migration of trial families and giving away or misuse of nets. Attrition due to reasons other than loss of integrity is a drain on the trial in terms of time and resources and creates the risk of leaving the trial underpowered for measuring true attrition due to loss of integrity. In some cases, losses to follow-up may make up 70% of the nets distributed at the start of a trial. Consequently, it is desirable to devise new procedures to limit such losses to follow-up.

How can net retention be improved? Any form of coercion would be unethical and impossible to enforce in practice. In the current WHO LLIN trial procedures, participating families are under no obligation to use or retain their nets. However, it might be possible to specify terms in the participant consent form that would help improve net retention while not affecting participants’ right to withdraw at any stage of the trial. In response to this issue, which arose from consideration of the present trial, the WHO proposed modifications to the consent forms used in Phase III trials and suggested the following new procedures:

### a) Cohort surveys

Study participants/families enrolled into the cohort component of the trial would be requested to consent to the following:

- Participants would not give away or sell the study nets;
- Participants would retain the freedom to stop using the nets at any time but should let investigators know the reasons when asked during the follow-up survey;
- Investigators would inform participants that the nets will be replaced after 3 years (at the end of the trial period and not before) regardless of net condition but only on production of the trial net which may be stored in the meantime for inspection;
- If participants stop using the trial net for any reason, including accumulation of holes, they must store the net for replacement after 3 years, or give it to the investigators who will replace it after the 3-year trial period has elapsed.

Such consent by participants would fulfil the needs of the trial and may reduce non-attritional losses, but would not affect participants’ right to stop using their nets at any time for whatever reason.

### b) Cross-sectional surveys

Other families who are eligible to be selected for cross-sectional surveys would have their nets replaced at the time of destructive sampling and would not be eligible for a second substitution at the end of the trial. Their consent form would be amended differently. They would be informed:

- That they should not give away or sell the study nets; and
- That they retain the freedom to stop using the nets at any time but are required to let investigators know the reasons when asked during the follow-up survey.

These were adopted by the WHO [19] and shall be included in the next edition of the WHO LLIN guidelines.

### Treatment of other polymer nets and materials

While this Phase III field trial evaluated the bio-efficacy and wash-fastness of ICON Maxx on polyester nets, a parallel Phase I study assessed ICON Maxx treatments on netting made of cotton, polyethylene, nylon, white and dyed polyester. The aim was to widen the range of household materials that vector mosquitoes may encounter in broader range of settings, and which could be rendered insecticidal without having to specify target product profiles or create bespoke products which may not justify the cost of investment as specific products or interventions.

Evaluation compared WHO cone, cylinder and tunnel tests using *An. gambiae*. ICON Maxx treated polyester and polyethylene netting met the WHO cone and tunnel test bio-efficacy criteria for LLIN after 20 standardized washes, and nylon and cotton netting passed the WHO tunnel test criterion of 80% mortality after 20 washes. The correlation of these findings with the current Phase III data on polyester raises the prospect of using ICON Maxx as an effective approach for converting untreated nets, curtains, military clothing, blankets, top-sheets and tents and tarpaulins, as used in disasters and humanitarian emergencies, into effective long-lasting insecticidal products for vector control of malaria [25–27], leishmaniasis [28] and dengue [29]. It may also provide a solution to the problem of reduced LLIN coverage between universal coverage campaigns by enabling conversion of commercially sourced untreated polyester and polyethylene nets into LLINs via community treatment. It may also prise open a new door to binding of non-pyrethroid insecticides to nets and textile materials for control of pyrethroid resistant vectors.

## Conclusion

This WHO Phase III household randomized trial of ICON Maxx treated polyester nets conducted in Tanzania achieved the combined cone and tunnel test efficacy criteria after 36 months of use. On the basis of this and other trials, and noting the overall bio-efficacy of the ICON Maxx long-lasting treatment for polyester nets, full recommendation was granted with an estimated duration of insecticidal efficacy of 36 months depending on the local setting. To guarantee efficacy, ensure proper net treatment and to minimize losses to reasons other than physical integrity, health education leaflets and packages should be provided concurrently with ICON Maxx sachet distribution.

## Data Availability

The datasets used and/or analyzed during the current study are available from the corresponding author on reasonable request.
